# Genetic Variation and Covariation in Male Attractiveness and Female Mating Preferences in *Drosophila melanogaster*

**DOI:** 10.1534/g3.113.007468

**Published:** 2013-11-08

**Authors:** Nicholas L. Ratterman, Gil G. Rosenthal, Ginger E. Carney, Adam G. Jones

**Affiliations:** * Department of Biology, Texas A&M University, College Station, Texas 77843; † Centro de Investigaciones Científicas de las Huastecas “Aguazarca,” Calnali, Hidalgo, México

**Keywords:** preference function, mate choice, sexual selection, Drosophila, genetic correlation

## Abstract

How mating preferences evolve remains one of the major unsolved mysteries in evolutionary biology. One major impediment to the study of ornament-preference coevolution is that many aspects of the theoretical literature remain loosely connected to empirical data. Theoretical models typically streamline mating preferences by describing preference functions with a single parameter, a modeling convenience that may veil important aspects of preference evolution. Here, we use a high-throughput behavioral assay in *Drosophila melanogaster* to quantify attractiveness and multiple components of preferences in both males and females. Females varied genetically with respect to how they ranked males in terms of attractiveness as well as the extent to which they discriminated among different males. Conversely, males showed consistent preferences for females, suggesting that *D. melanogaster* males tend to rank different female phenotypes in the same order in terms of attractiveness. Moreover, we reveal a heretofore undocumented positive genetic correlation between male attractiveness and female choosiness, which is a measure of the variability in a female’s response to different male phenotypes. This genetic correlation sets the stage for female choosiness to evolve via a correlated response to selection on male traits and potentially adds a new dimension to the Fisherian sexual selection process.

Despite the tremendous amount of progress that has been made in the field of sexual selection during the last four decades, the evolutionary dynamics of mating preferences remain one of the most challenging areas of study. Numerous empirical studies have addressed various aspects of ornament-preference coevolution (Bakker 1993; Wilkinson and Reillo 1994; Blows 1999; Gray and Cade 1999; Brooks and Endler 2001; Iyengar *et al.* 2002; Arnqvist and Kirkpatrick 2005; Qvarnström *et al.* 2006; Shaw and Lesnick 2009; Hohenlohe and Arnold 2010; Wiley and Shaw 2010; Wiley *et al.* 2012), but we still appear to be far from a consensus regarding the relative importance of various models of preference evolution. A major barrier to progress in this area stems from the problems associated with studying the genetic basis of preferences (Rodriguez *et al.* 2013), which tend to be complex and can only be quantified from large, labor-intensive studies (Wagner 1998; Chenoweth and Blows 2006; Jones and Ratterman 2009). Nevertheless, quantification of the genetic basis of preferences is a key requirement to test predictions of sexual selection theory because genetic correlations between ornaments contributing to attractiveness and behavioral phenotypes leading to mate choice play a central role in the most influential models of preference evolution (Arnold 1983; Kirkpatrick 1987; Heisler 1994; Mead and Arnold 2004).

Many of the problems associated with the study of mate choice become apparent from the seemingly simple exercise of defining terms. Although models usually describe mating preferences using a single variable for the sake of mathematical tractability, real mate choice is composed of multiple components, and probably cannot be fully described so simply (Brooks and Endler 2001; Rodriguez *et al.* 2013). Preferences can be visualized as a function with a mating response on the *y*-axis and ornament values that contribute to attractiveness on the *x*-axis (Figure 1). Most models of intersexual selection parameterize preference functions in one of two distinct ways. In one class of models, which are termed “open-ended preferences,” all individuals are assumed to prefer mates with the most extreme trait values, even though the magnitude of preferences may vary. This model predicts that all choosers will rank prospective mates in the same order but that some individuals will discriminate more strongly among mates than others (Figure 1A; Lande 1981). In the other main class of models, termed “unimodal preferences,” each chooser is assumed to show a peak preference for a particular mate phenotype, indicated by the ornament value that maximizes their mating response. Individuals may vary in the location of the peak (Figure 1C), the width of their preference function (Figure 1E), or both (Figure 1G), in which case two parameters would be necessary to describe a particular individual’s mating preferences.

**Figure 1:**
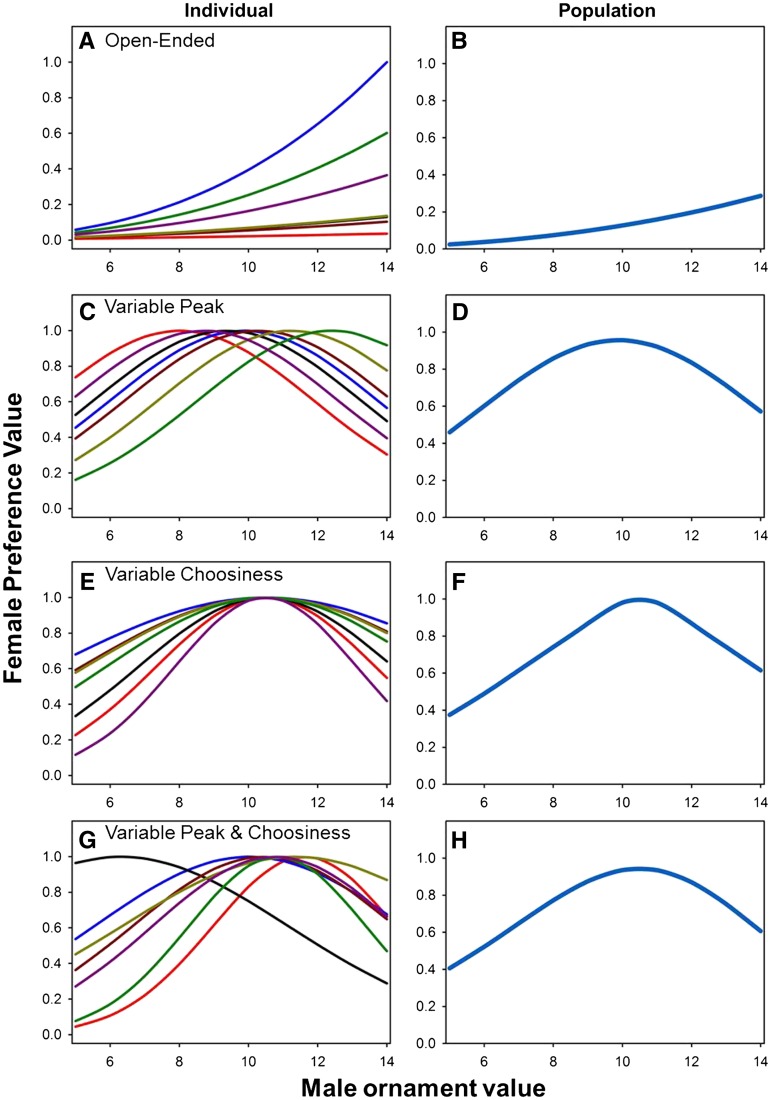
Results of simulations demonstrating different models of female preference functions as well as the relationship between individual-level preference functions (left column) and population-level preference functions (right column). We simulated populations of female flies and imposed a particular mating preference function on them. Then, we calculated each female’s probability of mating with individual males whose phenotypes were evenly spaced across the possible male trait values. The scales on both axes are arbitrary, so the general patterns are more important than any specific values. (A) and (B) show results from an open-ended preference function in which female preference is proportional to *e^yz^*, where *y* specifies the female’s preference steepness and *z* is the natural logarithm of the male’s trait value (Lande 1981). For this example, we drew values of *y* from a normal distribution with a mean of 2 and SD of 0.3. In (A), each curve shows the preferences of an individual female across 10 male phenotypes, and we show results for seven randomly chosen females. (B) shows the population-level preference function, calculated by averaging across 10 females. (C) through (H) are based on unimodal preference functions. In these panels, the left column also shows individual preferences functions for seven randomly chosen females, and the right column shows population-level preferences averaged across females. For the unimodal functions, female preference is proportional to $e^{-\frac{{\left(z-y\right)}^{2}}{2\nu^{2}}}$, where *z* is the male trait value, *y* is the female’s peak preference, and *ν* specifies the width of the female’s preference function. In (C) and (D), we held *ν* at 15 and drew each female’s *y* from a normal distribution with a mean of 10.5 and SD of 2. In (E) and (F), we drew *ν* from a normal distribution with a mean of 15 and SD of 7 while holding *y* constant at 10.5. In (G) and (H), we drew both *ν* and *y* from normal distributions with the same means and SDs used in the panels (C) through (F).

If we focus on the case of female choice among males for simplicity, we can see that several elements of female behavior are reflected in mating preference functions. For instance, “responsiveness” describes the mean response of the female across all males, a value that provides a window into female motivation to mate (Rowland *et al.* 1995; Reinhold *et al.* 2002; Bailey 2008). “Choosiness,” sometimes called discrimination (Brooks and Endler 2001), selectivity (Hedrick and Weber 1998), or preference strength (Fowler-Finn and Rodríguez 2012), is a measure of the variation in female responses to different phenotypes: choosier females are more variable in their responses to males differing in attractiveness, regardless of whether the model of preference is open-ended or unimodal (Gray and Cade 1999; Brooks and Endler 2001; Bailey 2008). We prefer the term “choosiness” because it is used primarily in the sexual selection literature in the context of mate choice, whereas other terms, such as “discrimination,” “selectivity,” and “preference strength,” are used to describe a wide range of phenomena across a number of disciplines outside of sexual selection research. Finally, if the preference function has an intermediate peak, then each individual female may have a “peak preference,” an ornament value to which she responds most readily. In principle, each female could have a different preference function, so females may show variation in responsiveness, choosiness, and peak preference (Figure 1) (Rowland *et al.* 1995; Gray and Cade 1999; Murphy and Gerhardt 2000; Brooks and Endler 2001; Reinhold *et al.* 2002; Bailey 2008; Rodriguez *et al.* 2013). It is also important to realize that although choosiness could be critically important in either model of preference functions, the most convincing empirical demonstrations of intersexual genetic correlations between mating preferences and sexually selected traits have focused on peak preference rather than choosiness (Wilkinson and Reillo 1994; Shaw and Lesnick 2009; Wiley and Shaw 2010; Wiley *et al.* 2012).

Here we investigate several unresolved aspects of mating preferences in *Drosophila melanogaster* by using isogenic lines to measure attractiveness and preferences. We take a different approach from many studies involving inbred lines by focusing on a detailed characterization of a small number of lines rather than a broader phenotyping effort involving dozens or hundreds of lines. Because we are interested in the shape of female preference functions and genetic variation in male attractiveness, we benefit more from precise estimates of preference functions from a small number of lines (as shown in Figure 2) than from imprecise estimates from a larger number of lines. With this constraint in mind, we set out to accomplish three specific goals. First, we quantify the extent to which aspects of mating preferences and sexual attractiveness in both males and females show a genetic basis. Second, we use these data to distinguish between the open-ended and unimodal models of male and female preference functions, which result in distinct predictions regarding the nature of genetic variation in peak preference and choosiness (Figure 1 and Supporting Information, Figure S2). Finally, we test the widespread expectation from models of intersexual selection that mate choice should produce a genetic correlation between male attractiveness and aspects of female preferences.

**Figure 2:**
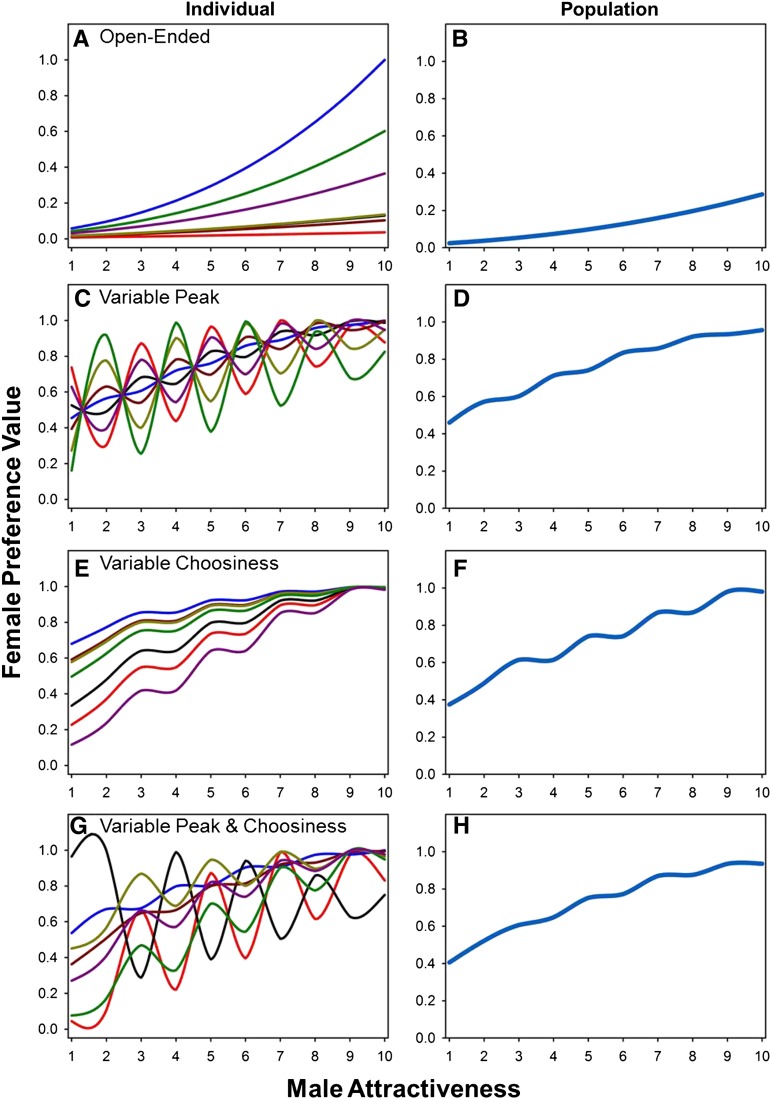
Individual-level (left column) and population-level (right column) female preference functions for overall male attractiveness. These figures are based on the same simulated data used in Figure 1, except now we assume that we do not have access to male trait values. Males are instead ranked on the basis of their average attractiveness to 10 randomly chosen females, with greater ranks being more attractive (*i.e.*, 1 is least attractive and 10 is most attractive). Regardless of the shape of individual-level preference functions, the population-level preference function for male attractiveness is open-ended by definition (right column). However, an examination of individual-level female preferences for males of varying average attractiveness shows that different types of individual-level preference functions (see Figure 1 for the shapes of the preference functions for the underlying male trait) produce very different patterns of among-female preferences. In particular, when peak preferences vary, different females show different rank-order preferences for males. Hence, data regarding female preferences for overall male attractiveness can shed light on the underlying preference functions for the traits involved in sexual selection (see supplemental File S1 for a more in depth discussion of these issues).

## Materials and Methods

### Inbred lines, controlled larval density, and general culturing procedures

Ten randomly chosen inbred lines from the Drosophila genetic reference panel (RAL-208, -304, -315, -360, -379, -437, -486, -517, -707, and -732) were obtained from the Bloomington Drosophila Stock Center (Bloomington, IN). The lines were cultured in 8-dram vials with approximately 10 mL of cornmeal-molasses-agar medium (1 L of water, 30 g of nutritional yeast extract, 55 g of cornmeal, 11 g of Droso-agar, 72 mL of molasses, 6 mL of propionic acid, and 16 mL of 15% tegosept in ethanol) seeded with 2 drops of yeast suspension (1 g of live yeast, 333 µL of 1% acetic acid, 5 mL of water) at 25° (±2°), 60% relative humidity (±3%) on a 12:12-hr light cycle.

Lines were cultured under controlled larval density for at least two generations before testing in an effort to reduce environmentally induced phenotypic variation. To control for larval density, we used juice-agar plates with a smear of yeast paste (1 g of active dry yeast, 1.3 mL of water) to collect first instar larvae. Twenty individuals of each sex were left in laying pots (juice-agar plates and inverted plastic beakers) for 24 hr. Plates were changed each day for 3 d. First instar larvae were picked with toothpicks and placed in 10 mL of food at a density of 50 larvae per vial. Eclosing adults were collected every 12 hr using CO_2_ anesthesia. Adults from the F_2_ generation and later were used in behavioral assays as well as for starting the next generation of controlled larval density. Individuals used for the behavioral assays were separated by sex into vials with 5 mL of food with five individuals per vial. All flies were 3−6 d old at the time of testing. Vials containing females were retained after the flies were assayed to assure virginity. Any trials containing a female from a nonvirgin vial were discarded from the analysis. We randomized both the ages of flies, within the 3-to 6-d age window, and the days on which we conducted mating assays for particular pairwise tests. In other words, each pairwise test between lines was replicated (at least 10 times), and each replicate was run on a randomly chosen day with flies of random age. This approach controls for possible effects of environmental variables that change as a function of variable weather patterns or fly age.

### Mating arrays

We designed a new type of mating apparatus for the behavioral assays. These mating arrays were designed to facilitate high-throughput testing and to allow males and females to be acclimated separately until each trial started. This method allows 20 no-choice tests to be conducted simultaneously. Each array consisted of 20 mating chambers (Figure S1) measuring 1 inch in diameter and arranged in four rows of five columns. Arrays consist of four 12.5 × 6.5 × 0.125 inch pieces (layers) of PETG plastic (SABIC Polymershapes, Jessup, MD) and a track that holds the four layers in place, allowing each piece of plastic to slide back and forth independently. The outer layers act as physical barriers to keep the flies in the chambers (*i.e.*, floors and ceilings). Each outer layer has 20 entry holes slightly larger than the tip of the aspirator for flies to be loaded into the chambers. The middle two pieces comprise the mating chambers. The cylindrical chambers are 1 inch in diameter (identical to the vials in which the flies are housed) and 0.125 inches in height. When the two middle layers are aligned (Figure S1, C and D) the depth of the mating chamber doubles to 0.25 inches. See Figure S1 for more details.

### Mating assays

Trials were run from 0 to 2 hr after the lights turned on each day. All trials were conducted in the same environmentally controlled room where the flies were cultured. Virgin males and females were aspirated singly into each chamber when the mating array was in the out-of-phase position (Figure S1A). Males and females were loaded on each layer separately, which was alternated for each array. For example, all males would be loaded on the upper layer first, followed by females on the lower layer. The next array would have females loaded first (upper layer) and males loaded second (lower layer). The loading of each array took approximately 20 min. We recorded the order in which each individual was loaded to statistically control for order effects if they were present, which they were not (data not shown).

Fully loaded arrays were placed on a light box (Model 4 Slide Sorting Viewer, Graphic Technology, Inc., Newburgh, NY) to provide illumination from beneath the arrays. After 10 minutes of acclimation, recording began from a video camera (JVC GZ-HD300BU) mounted directly above the light box and the chambers were aligned by sliding the two pieces of plastic until the two chambers became one (Figure S1, C and D). All trials were recorded for 1 hr. A light diffuser (8’ × 4’) was suspended above the cameras and light box to reduce reflections and inconsistent lighting from the fluorescent lights in the room.

Each video was scored for courtship latency, copulation latency, and copulation duration. Courtship latency was measured as the time from aligning the chambers to male orientation toward the female. Copulation latency was measured as the time from the onset of courtship to the male mounting the female. Copulation duration was measured as the time from mounting to separation of the pair. Each pair was given a score depending on whether they courted and a separate score depending on whether or not they mated.

A fully factorial design was employed: there were 100 possible pairings between the 10 genotypes. Each of the 100 possible pairings was replicated enough times to obtain at least 10 successful matings, although not all pairs mated. Some pairs had a low percentage of trials that resulted in mating; therefore, the total sample size of tests varied. For example, the pairing between males from line 208 and females from line 304 had a total sample size of 11: one pair failed to mate. The sample sizes for each pairing range from 10 to 27, meaning that the proportions of successful matings range from 0.37 to 1.0. In some cases, we obtained more than 10 matings, because we occasionally set up more pairings than the bare minimum necessary to obtain the desired 10 successful matings. In these cases, we included all the resulting data in our statistical analyses. A total of 1322 trials were conducted in 71 arrays (not all arrays were full, and some individuals were damaged during the experiment and discarded).

### Statistical analysis

We recorded three scores for each pair: courtship latency, copulation latency, and copulation duration. Means and SDs were measured separately for each sex of each line. With respect to courtship latency and copulation duration, which measure male-controlled components of courtship and mating (Gilchrist and Partridge 2000; O’Dell 2003), an effect of male genotype indicates genetic variation in male preference components, whereas an effect of female genotype indicates genetic variation in female attractiveness. Conversely, for copulation latency, which is controlled by females in *D. melanogaster* (females actively reject males and thus demonstrate active mate choice; Connolly and Cook 1973), a female genotype effect signals genetic variation in female preference, and a male genotype effect indicates genetic variation in male attractiveness. Copulation duration calculations exclude data from non-mating pairs.

Following Brooks and Endler (2001), we analyzed three components of the choice data. For the following explanations, we are specifically describing data related to the choosing sex. For example, preference components were measured on males for latency to court and copulation duration. Similarly, measures of female copulation latency were used to estimate female preference components. Each line’s mean value quantifies responsiveness and the SD of the mean quantifies choosiness. As choosiness can be calculated multiple ways [*e.g.*, coefficient of variation in response (Hedrick and Weber 1998); coefficient of variation in response squared (Fowler-Finn and Rodríguez 2012); difference between maximum response and mean response divided by total SD (Gray and Cade 1999); difference between maximum response and mean response divided by SD in maximum response (Bailey 2008)], we chose to use the SD in response (Brooks and Endler 2001) as it is the most straightforward measure (note that Brooks and Endler refer to this phenotype as “discrimination,” although both measures are identical).

The distributions of courtship latency and copulation duration were right skewed, so we compared the plots of the residuals against the predicted values for raw and log transformed data. In both cases the plots assumed a more circular shape when in the log form, thereby justifying the transformation (Quinn and Keough 2002). Therefore, each response variable was transformed to the natural logarithm before the analysis. We used separate general linear models to analyze the courtship latency and copulation duration data. For copulation latency we used a Cox proportional hazards model because the data were right censored (*i.e.*, there were pairs that did not mate in the allotted time of one hour) (Moeschberger and Klein 2003). For each response variable (courtship latency, copulation latency, and copulation duration), we built a model with male genotype, female genotype, male age, female age, and male genotype × female genotype interaction, which we refer to as the base model. For copulation latency, we included courtship latency as an additional factor in the model (*i.e.*, the base model effects plus courtship latency). Similarly, for copulation duration we added courtship latency and copulation latency as effects (*i.e.*, the base model effects plus courtship and copulation latencies) to control for their effects.

Because we measure choosiness as a SD among means (*i.e.*, each line has a mean courtship latency, copulation latency, and copulation duration with each of the other ten lines and the SD among these means is the choosiness), we used Levene’s test to compare the corresponding variances among lines. If lines differ significantly with respect to variance, then they also differ with respect to SD. Because the lines are isogenic, significant variation among lines signifies the existence of genetic variation for the trait of interest.

As mentioned previously, each of the 100 possible pairings was selected to occur at a random time (within the 2-hr window) on a randomly chosen day. We recorded the day, time of day, position of each pair on the array, humidity, and temperature for each pair of individuals. These factors were included in a preliminary analysis and were found to have no effect on any of the response variables (courtship latency, copulation latency, copulation duration), so we removed them from the models.

We controlled for multiple tests by using the false-discovery rate method outlined in Benjamini and Hochberg (1995), although none of the results were affected. All statistics were performed in JMP 9 (SAS Institute, Inc., Cary, NC). Our raw data are available in supplemental File S2, and we present means and standard deviations for the preference variables for all crosses in supplemental Table S1, Table S2, and Table S3.

## Results

Our first goal was to test for the existence of genetic variation in components of mate choice for both sexes in *D. melanogaster*. Statistically significant differences in mean preference values among lines demonstrated that flies exhibit detectable genetic variation for all three types of mating preference (male courtship latency, female copulation latency, and male copulation duration) as well as for male and female attractiveness (see significant male and female genotype effects in Table 1, Table 2, and Table 3).

**Table 1:** Copulation latency is affected by male and female genotype, as well as the interaction between genotypes

Source	df	Likelihood Ratio χ^2^	*P*-Value
**Male genotype**	**9**	**71.644**	**<0.0001**
**Female genotype**	**9**	**337.892**	**<0.0001**
Male age	4	5.892	0.207
Female age	3	18.893	0.0003
**Male × female genotype**	**81**	**140.248**	**<0.0001**
Courtship latency	1	0.004	0.9489

A model explaining copulation latency was built that included sex-specific genetic effects, age effects, and the effect of courtship latency. Genetic variation exists for both males and females for precopulatory attractiveness (males) and precopulatory preferences (females). The significant interaction indicates that combinations of genotypes contribute to variation in copulation latency as the result of different rank-ordering of males by females.

**Table 2:** Courtship latency is affected by male and female genotype

Source	df	Sum of Squares	Mean Square	F-Ratio	*P*-Value
Model	106	282.480	2.769	3.006	< 0.0001
Error	1215	1115.409	0.918		
Total	1321	1407.889			
**Male genotype**	**9**	**160.584**	**17.843**	**19.436**	**< 0.0001**
**Female genotype**	**9**	**40.855**	**4.540**	**4.945**	**< 0.0001**
Male age	4	2.122	0.530	0.578	0.679
Female age	3	0.793	0.264	0.288	0.834
Male × female genotype	81	76.371	0.943	1.027	0.416

A model explaining courtship latency was built that included sex-specific genetic effects, and age effects. Both male and female genotypes contribute to variation in courtship latency, demonstrating genetic variation in male courtship propensity and female precopulatory attractiveness.

**Table 3:** Copulation duration is influenced by male and female genotype

Source	df	Sum of Squares	Mean Square	F-Ratio	*P*-Value
Model	108	28.650	0.265	2.178	< 0.0001
Error	860	104.760	0.122		
Total	968	133.410			
**Male genotype**	**9**	**8.330**	**0.926**	**7.599**	**< 0.0001**
**Female genotype**	**9**	**3.711**	**0.412**	**3.385**	**0.0004**
Male age	4	0.990	0.248	2.032	0.0880
Female age	3	1.437	0.479	3.931	0.0084
Male × female genotype	81	8.617	0.106	0.873	0.777
Courtship latency	1	4.902	4.902	40.239	< 0.0001
Copulation latency	1	0.561	0.561	4.608	0.0321

We built a model to explain variation in copulation duration due to sex-specific genetic effects, age effects, courtship latency, and copulation latency. Genetic variation exists for female postcopulatory attractiveness and male copulatory duration, but we see no evidence for a male genotype × female genotype interaction.

### Genetic variation in behavioral components of mate choice: females

**Figure 3:**
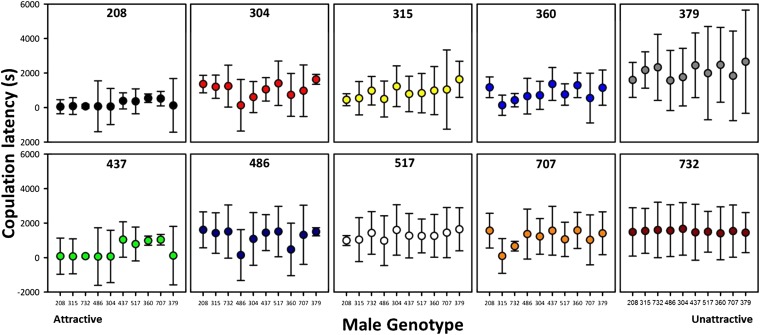
Individual female preferences for each isogenic line. Male genotypes have been ordered according to their global attractiveness along the x-axis and the orders are identical in each graph, with the most attractive genotype (208) on the left and least attractive genotype (379) on the right. Copulation latency is measured in seconds and the mean ± SD is reported for each pairing between lines. Note that lower y-values indicate greater attractiveness because less time is needed for those males to attain copulations. Responsiveness is measured as the mean copulation latency of females from each genotype and varies across lines (*e.g.*, 208 *vs.* 707). Choosiness is measured as the SD of the mean (responsiveness), with more variability in responses (*e.g.*, 486) being considered choosier than less variable responses (*e.g.*, 707 is choosier than 732).

To investigate the variation in female preferences among lines in more detail, we ranked male lines according their average attractiveness across all females (each genotype’s mean male copulation latency) and plotted the responses of females from each genotype separately (Figure 3). Several important observations emerge from this analysis. First, mean copulation latency for females varies substantially across lines, indicating genetic variation in responsiveness. Thus, females from some genotypes require more stimulation prior to mating than others, regardless of the identity of her potential mate. Second, we found genetic variation among lines in choosiness (calculated as the SD in female copulation latency across males from all ten genotypes; Levene test: F_9,1312_ = 2.6431, *P* = 0.0049). Thus, responsiveness and choosiness exhibit genetic variation and consequently have the potential to respond to selection and to be genetically correlated with other traits, such as male attractiveness.

The shapes of the female preference functions for overall male attractiveness varied considerably among lines (Figure 3). Importantly, Figure 3 shows that some female lines ranked males in terms of attractiveness differently than other female lines, and these differences were statistically significant (see SD bars in Figure 3 and significant male genotype × female genotype interaction in Table 1). This sort of differential ranking occurs in unimodal preference functions when the position of the peak varies, but does not occur in open-ended preference models (Figure 2) or unimodal functions with no inter-individual variation in the location of the peak preference. We can conclude from these observations that female *D. melanogaster* show unimodal preference functions with genetic variation in peak preferences (or possibly a preference function even more complex than those considered here).

### Genetic variation in behavioral components of mate choice: males

Our results for male preferences are similar to those for females in the sense that we see evidence for significant genetic variance in both male courtship latency (precopulatory responsiveness: Table 2) and copulation duration (postcopulatory responsiveness: Table 3). Courtship latency can be interpreted as a measure of male motivation to mate, and the significant effect of male genotype (Table 2; *P* < 0.0001) shows that *D. melanogaster* populations harbor genetic variation for this trait. A significant effect of female genotype on courtship latency (Table 2; *P* < 0.0001) further indicates that some female genotypes are more attractive to males than others, and are thus courted faster. We found a similar pattern for copulation duration (Table 3; male genotype: *P* < 0.0001, female genotype: *P* = 0.0004), which suggests that *D. melanogaster* populations are characterized by genetic variation in male responsiveness and female attractiveness to males during both pre- and postcopulatory phases of sexual selection.

When we turn our attention to male preference functions, we find a pattern that contrasts with female preferences. Specifically, we find no evidence for a male genotype by female genotype interaction for either courtship latency or copulation duration (Table 2, courtship latency interaction, *P* = 0.416; Table 3, copulation duration interaction, *P* = 0.777). Thus, males from all 10 genotypes tended to agree on which females were most attractive, so the male data are more consistent with an open-ended model of preferences or a unimodal function in which all males share the same peak preference (Figure 2). We compared pre- and postcopulatory male choice to see if these two phases of male choice were reinforcing or antagonistic and found no genetic correlation between female pre- and postcopulatory attractiveness (Figure S2), although we had limited power to detect such a relationship with only 10 lines in our study. This observation raises the possibility that males evaluate independent sets of traits during pre- and postcopulatory mate choice.

### Intersexual genetic correlations

**Figure 4:**
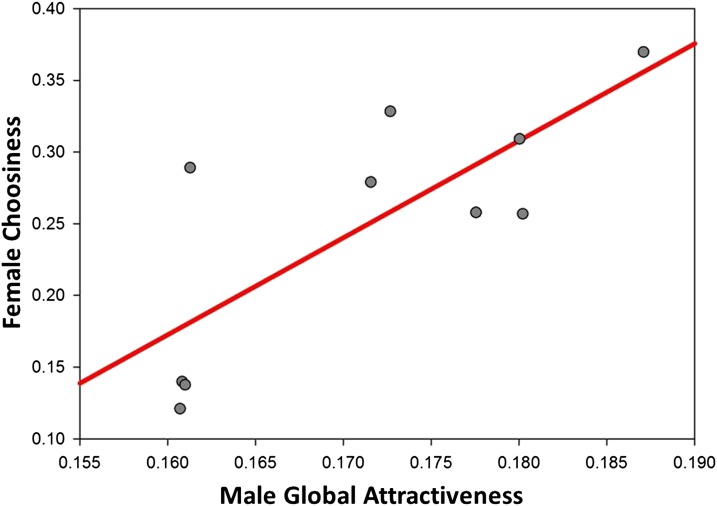
A positive genetic correlation between male attractiveness and female choosiness. We estimated the genetic correlation by regressing the SD in female copulation latency (choosiness) on mean male copulation latency (global attractiveness) for all ten isogenic lines. The relationship indicates that genotypes with heightened male attractiveness also have increased female choosiness (r^2^ = 0.597; n = 10; *P* = 0.008). For the purpose of demonstrating the positive nature of the genetic correlation, we used inverse copulation latency in seconds so that larger values of male attractiveness corresponded to more attractive males. The range of values for males is different than that for females, because male global attractiveness is a mean, whereas female choosiness is a SD.

The final goal of our study was to test the prediction from models of intersexual selection that populations should evolve a genetic correlation between aspects of male attractiveness and aspects of female preference (Lande 1981; Kirkpatrick 1987; Heisler 1994; Mead and Arnold 2004). Our results are consistent with this prediction in the sense that we detected a positive genetic correlation between male attractiveness and female choosiness (Figure 4). Thus, genotypes resulting in attractive males also result in choosy females, whereas genotypes with indiscriminate females tend to have males considered unattractive to the average female across all genotypes. This result is predicted by verbal models exploring variation in choosiness (Widemo and Sæther 1999), because females that are more discriminating in their mating decisions are more likely to obtain their most-preferred mate. Conversely, nonchoosy females that do not discriminate strongly among males are more likely than choosy females to mate with globally unattractive males. In short, our results support the idea that mate choice in *D. melanogaster* produces a genetic correlation between male attractiveness and female choosiness and that choosiness has the potential to evolve as a result of a correlated response to selection on male sexually selected traits that contribute to attractiveness.

## Discussion

Our study demonstrates several important, novel findings regarding mating preferences in *D. melanogaster*. Interestingly, this species appears to exhibit sexual dimorphism with respect to mating preference functions. In particular, males are characterized by preference functions in which different male genotypes largely rank females similarly by attractiveness, a situation that arises under open-ended preference functions or unimodal preference functions with no genetic variation in peak preferences. Females, on the other hand, show a very different pattern: they likely exhibit unimodal preference functions with variation among females in both peak preferences and choosiness (or possibly even more complex mating preference functions of yet to be determined shape). This situation results in different female genotypes ranking males differently in terms of attractiveness as well as exhibiting genetic variation in terms of their responses to males from the 10 lines. Our final major result involved the detection of a previously undocumented genetic correlation between female choosiness and male attractiveness, which could facilitate a correlated response to selection in choosiness as male traits evolve via sexual selection.

The observation that males from different genotypes generally agreed on the attractiveness of females, while females varied in their rank-order preferences, could be attributed to a number of possible causes. For instance, if females attend to several different aspects of the male phenotype in making mating decisions, then it may be possible for different males to achieve similar levels of average attractiveness despite different underlying combinations of trait values (Blows *et al.* 2003). For instance, if females weigh various male traits even slightly differently when they decide whether to mate with a male, then we might expect the males that are most attractive on average to sometimes be considered average or unattractive by certain females in the population. This sort of situation would be especially likely if both directional and stabilizing selection on female mating preferences tended to be weak, which would allow the accumulation of substantial genetic variation in preferences within populations. In addition, if mating preferences are highly polygenic, then they could harbor a large amount of genetic variation by virtue of being a large mutational target. Why male genotypes appeared to rank female genotypes in the same order in terms of attractiveness is a separate but related question. One possibility is that males prefer traits tied to female fecundity, which could provide a direct benefit for male mate choice, especially under conditions where sperm limitation is a possibility (Edward and Chapman 2011). Thus, males preferring more fecund females would obtain direct fitness benefits, which would cause these preferences to increase in frequency in the population. Given a sufficiently large fitness benefit in terms of additional offspring, we might expect an open-ended preference for fecund females to come to predominate in the population. A second possibility is that our sample size or study design was simply not sufficient to demonstrate a statistically significant interaction between male and female genotypes for the aspects of male preferences that we measured. Regardless, our results imply that males exhibit less genetic variation for preference functions, especially in terms of the rank-order of prospective mates, compared to females in *D. melanogaster*.

### Implications for sexual selection theory

Analytical theory has traditionally collapsed mating preferences into a single parameter as a modeling convenience, despite the possibility that mating preference functions can be complex (see Heisler 1994). Our results indicate that female preference functions probably vary in terms of peak preference (*i.e.*, the phenotype of the most-preferred male) and choosiness (*i.e.*, the width of the preference function), so at least two parameters should be necessary to describe mating preferences in female *D. melanogaster*. Our results also indicate that choosiness is genetically correlated with male attractiveness. Moreover, our experimental design allowed us to infer that female preferences have a unimodal shape, such that each female is expected to have a unique peak-preference that describes her preferred male phenotype. However, without access to multivariate male phenotypes incorporating all possible phenotypic characters involved in mate choice (some of which are almost certainly impossible to measure), we were not able to identify the locations of these peaks in phenotypic space. Thus, our results leave open the possibility that peak preferences also are genetically correlated with male attractiveness or specific male trait values. Future work could profitably explore such correlations, and this goal will require the dissection of overall male attractiveness into its multivariate phenotypic components.

As noted previously, existing theory predicts a genetic correlation between ornament values in males and the slope of the preference function (for open-ended preferences) or the peak preference (for unimodal preference functions) (Lande 1981; Heisler 1985; Hall *et al.* 2000). We extended this robust result to a new behavioral phenotype, choosiness, which describes the degree of discrimination among potential mates and can be independent of the location of the peak preference in unimodal preference functions (Gray and Cade 1999; Brooks and Endler 2001). Our observations are consistent with those obtained from analytical theory (Lande 1981; Heisler 1985; Hall *et al.* 2000): females with larger than average variation in their responses to males (choosy females) tend to mate with more attractive males, resulting in a genetic correlation between male attractiveness and female choosiness. This genetic correlation provides the foundation for Fisherian sexual selection to operate but is peculiar in that it combines attributes of the preference functions that differ from those normally used to model mate choice. This result suggests that existing single-parameter models of preference evolution may be inadequate to capture the complexity of intersexual selection in natural populations. Considering the evidence for genetic variation in multiple components of choice behavior and an intersexual genetic correlation, these results call for more explicit models of female mating behavior to investigate which specific attributes of mate choice are most likely to coevolve with male attractiveness.

### Caveats

Several limitations of our study call for caution in drawing broad generalities from our observations. The fact that we used inbred lines almost certainly had some sort of effect on mating preferences and male attractiveness. We would predict that inbred males would be less attractive on average compared with outbred males in a sexually reproducing species like *D. melanogaster*. In addition, highly inbred females could display mating preferences different from those that typically occur in wild-type females. However, we chose to use inbred lines for two reasons. The first is that the inbred lines that comprise the *D. melanogaster* Genetic Reference Panel (Mackay *et al.* 2012) are currently being characterized with respect to a vast array of phenotypic traits, including many that are relevant to male attractiveness and sexual selection. Even though individuals from each line are severely inbred and may possess traits that rarely occur in nature, the comparison of individuals across lines nevertheless provides valuable information regarding the genetic basis of quantitative traits (Ober *et al.* 2012; Harbison *et al.* 2013). The second reason that we did not shy away from using inbred lines is that the study of sexual selection and mating preferences in *Drosophila* has a long history in which the benefits of using inbred lines has often outweighed the costs. For instance, in the very earliest days of sexual selection research, Bateman (1948) used inbred lines for his classic experiments, and more recent studies specifically dealing with male attractiveness and female preferences in *Drosophila* have continued to use inbred lines to gain important insights (Sharma *et al.* 2010; Ingleby *et al.* 2013). These studies of inbred lines have allowed us to gain an understanding of what is possible and a rough idea of the genetic underpinnings of the sexual selection process. However, future studies of natural populations or crosses between lines will no doubt play a critical role in refining our current conceptualization of sexual selection in *D. melanogaster*.

A final caveat is that secondary sexual traits have a tendency to be condition dependent (Rowe and Houle 1996), although our experimental design controlled for environmental variation as much as possible. When attempting to isolate genetic effects on phenotypic variation, it is useful to raise individuals in a standardized environment to control for environmentally-induced phenotypic effects (Falconer and Mackay 1996). The goal of our study was not to look for condition dependent sexual selection in *D. melanogaster*. Instead, we sought to identify the genetic underpinnings of these interesting phenotypes. It is possible that condition affects both ornaments and preferences in the source population from which our study lines were derived, and our study serves as a stepping stone for future studies aimed at addressing this hypothesis.

In summary, this study reveals several novel and important aspects of the genetic basis of attractiveness and aspects of mating behavior in *D. melanogaster*, and many of our observations carry implications for the study of sexual selection in general. Importantly, we demonstrate that attractiveness and preferences of both males and females have a substantial genetic component, a result that agrees well with many other studies of the genetic basis of traits involved in sexual selection in a wide range of taxa (Merilä and Sheldon 1999; Rodriguez *et al.* 2013). Even though previous studies have shown that attractiveness has a genetic basis, our investigation of female mating preferences provides unprecedented insights into the nature of preference functions. Our data show that female *D. melanogaster* likely show unimodal preference functions and that genotypes differ from one another with respect to both peak preference and choosiness. Thus, theoretical models that collapse mating decisions into a single parameter provide an inadequate description of female preferences. Future theoretical and empirical work should consider the possibility that both peak preferences and choosiness may simultaneously evolve in nature. In addition, our results show that female choosiness is genetically correlated with male attractiveness, indicating that the genetic architecture of sexually selected traits and preferences in *D. melanogaster* is compatible with a Fisherian process of runaway sexual selection. Thus, our results provide a new perspective on the nature of sexual selection in general, and mating preference functions in particular, whose complexity will have to be embraced in future studies of the genetic underpinnings of attractiveness and preferences.

## Supplementary Material

Supporting Information
