# Supplementary material for: Genetic Variation and Covariation in Male Attractiveness and Female Mating Preferences in Drosophila melanogaster
Source: G3 (Bethesda). 2013 Nov 8;4(1):79–88. doi: 10.1534/g3.113.007468 (PMC3887542; doi:10.1534/g3.113.007468)
Supplement: Supporting Information [file supp_4_1_79__index.html]

Genetic Variation and Covariation in Male Attractiveness and Female Mating Preferences in Drosophila melanogaster — Supporting Information 

# Genetic Variation and Covariation in Male Attractiveness and Female Mating Preferences in *Drosophila melanogaster*

## Supporting Information for Ratterman *et al.*, 2014

**Files in this Data Supplement:**

- Supporting Information - Figures S1-S2, Files S1-S2, and Tables S1-S3 (PDF, 577 KB)
- Figure S1 - High-throughput mating arrays. (PDF, 394 KB)
- Figure S2 - Comparison of pre- and postcopulatory male mate choice. (PDF, 326 KB)
- File S1 - Supplementary Information (PDF, 311 KB)
- File S2 - Raw data file (.xlsx, 121 KB)
- Table S1 - Copulation latency means plus / minus standard deviations for each pairing (.xlsx, 10 KB)
- Table S2 - Courtship latency means plus / minus standard deviations for each pairing (.xlsx, 10 KB)
- Table S3 - Copulation duration means plus / minus standard deviations for each pairing (.xlsx, 10 KB)
