# Supplementary material for: Genetic Variation and Covariation in Male Attractiveness and Female Mating Preferences in Drosophila melanogaster
Source: G3 (Bethesda). 2013 Nov 8;4(1):79–88. doi: 10.1534/g3.113.007468 (PMC3887542; doi:10.1534/g3.113.007468)
Supplement: Supporting Information [file supp_g3.113.007468_FigureS1.pdf]

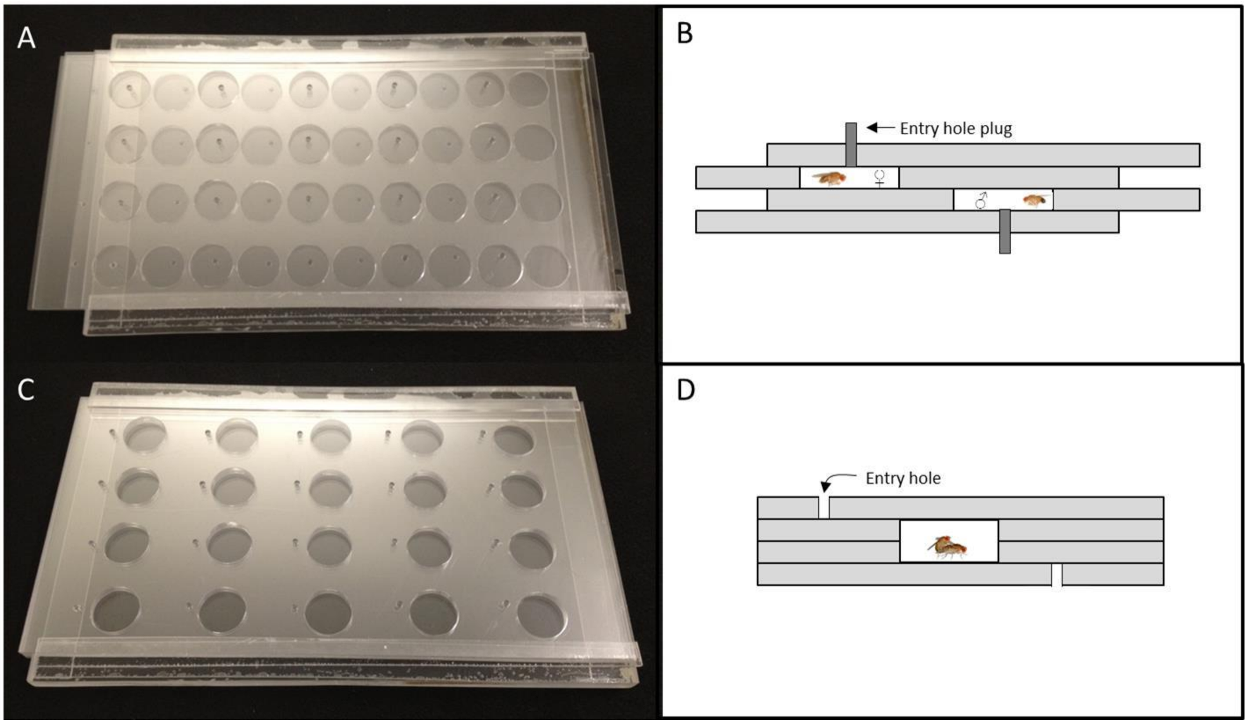

Figure S1 High-throughput mating arrays. Each array consists of four layers of plastic: a top and bottom sheet to keep the flies in the mating chamber, and two inner sheets to house the flies. (A) Out-of-phase position. Individuals were aspirated into each half of a chamber, one sex per side, alternated throughout the experiment. Thus, twenty males were contained in chambers separated from twenty females housed in separate chambers. (B) Cross-section of a single chamber while out-of-phase. Plugs were used to keep flies from escaping after they were aspirated into their individual chambers. After the final individual was loaded, the mating array was moved on top of a light source, underneath a video camera. Five minutes of acclimation were allowed, at which point the chambers were aligned by gently sliding the pieces of plastic so that each male and female were simultaneously introduced into one single chamber. (C) In-phase position. (D) Cross-section of a single chamber while in-phase. Pairs were given one hour to mate. If, at the end of an hour, any pairs were observed to be copulating, the video was allowed to continue recording until the male dismounted.
