# Supplementary material for: Genetic Variation and Covariation in Male Attractiveness and Female Mating Preferences in Drosophila melanogaster
Source: G3 (Bethesda). 2013 Nov 8;4(1):79–88. doi: 10.1534/g3.113.007468 (PMC3887542; doi:10.1534/g3.113.007468)
Supplement: Supporting Information [file supp_g3.113.007468_FigureS2.pdf]

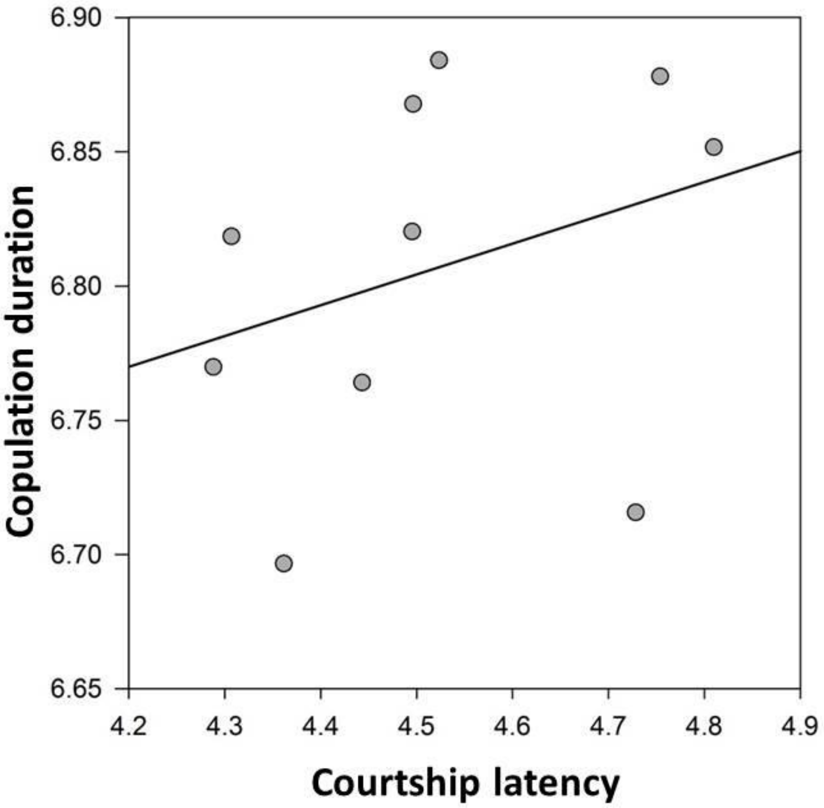

Figure S2 Comparison of pre- and postcopulatory male mate choice. No relationship was found: females that were courted sooner were not mated longer (ANOVA: $F_{1,8} = 0.9035$, $p = 0.370$). Units on both axes are measured on a log scale (ln(seconds)). Because short courtship latency and long mating duration are signs of male preference, a negative relationship would be expected in this plot if female genotypes that are attractive to males in precopulatory sexual selection are also attractive to males in the postcopulatory phase.
