# Supplementary material for: Genetic Variation and Covariation in Male Attractiveness and Female Mating Preferences in Drosophila melanogaster
Source: G3 (Bethesda). 2013 Nov 8;4(1):79–88. doi: 10.1534/g3.113.007468 (PMC3887542; doi:10.1534/g3.113.007468)
Supplement: Supporting Information [file supp_g3.113.007468_FileS1.pdf]

File S1

Supplementary Information

We identified two problems with measuring individual mating preferences. The first is that the characterization of individual-level mating preferences requires repeated measurements of the same female. If a female's preferences change over time or if she becomes habituated to the preference assay, then her true preferences may be nearly impossible to measure. The second problem is that there may be no way to know *a priori* which ornamental traits of males are most important in terms of mating decisions. Thus, if key traits remain unmeasured, then individual-level preference functions may prove inaccessible.

One approach to overcome the first problem is to take advantage of isogenic lines, in which all individuals are genetically identical. This approach allows separate females from the same line to be measured once each, removing any complications that could arise from repeated testing of the same individual. In addition, the mean response of multiple females from the same isogenic line with the same set of stimuli provides a measure of the genetic value (or breeding value) of the preference function for the isogenic line of interest. Thus, the use of isogenic lines (or clones if they happened to be available) provides one avenue to access the genetic preference function of a particular genome-wide genotype.

To circumvent the second issue, which occurs when the male traits that determine female preferences are unknown or infeasible to measure, it is possible to use a measure of overall male attractiveness rather than individual male trait values. We believe the problem of unmeasured traits is pervasive, even in systems in which some traits have been shown to be involved in mate choice, because female preferences in most complex organisms almost certainly involve the assessment of multiple traits across multiple sensory modalities (Candolin 2003; Partan and Marler 2005). For instance, *D. melanogaster* employs chemical, acoustic, visual, tactile, and gustatory signals during courtship, resulting in a potentially high dimensionality of mate choice (Hohenlohe & Arnold 2010). Most studies, however, focus on a single trait or multiple traits within the same modality for the very simple reason that even these studies are extremely labor intensive (Ritchie 1996; Blows *et al*. 2004). A measure of overall male attractiveness can be seen as a composite measure of trait values, filtered through the sensory and neurological machinery of the choosing females (Markow 1996; Ingleby et al. 2013). This view of preferences also has the added benefit of providing a framework in which to assess whether or not male attractiveness has a genetic basis, an important consideration for an evolutionary response to sexual selection (Wedell and Tregenza 1999).

It is one thing to suggest that male attractiveness is a useful property and quite another to devise a meaningful measure of male attractiveness. One possible approach is to measure attractiveness by the mean response of females to males of a particular genotype. Like the study of female preferences, this endeavor is facilitated by the availability of isogenic lines. The most attractive males from a particular genotype can be defined as the one to which females respond most readily on average and the least attractive male genotype is the one with whom females are most reluctant to mate (Brooks 2000; Ingleby et al. 2013). By repeatedly testing males from a given isogenic line across a panel of female genotypes, an average attractiveness score can be established for each male genotype.

Returning to our goal of characterizing female preference functions, we now must address the question of what preferences for male attractiveness can tell us about the underlying preference functions for male traits. In Figs. 1 and 2, we present simulated

data to illustrate the relationship between female preferences for particular male traits and female preferences for overall male attractiveness. This exercise produces some simple expectations for different models of female preference functions and provides a basis for identifying which model is most appropriate for a particular species in the absence of measurements of male ornamental values. At the population-level, of course, preferences for male attractiveness are open-ended by definition, regardless of the underlying female preference functions for traits (compare the right columns of Figs. 1 and 2). However, inspection of individual-level preference functions based on overall male attractiveness can provide insights into the particular model of female preferences. In the left column of Fig. 2, we show a subset of individual-level female preference functions for ten male genotypes ranked along the x-axis from least to most attractive. One important observation is that when females display open-ended preferences they invariably rank the males in the same order in terms of attractiveness, even though the females may differ in choosiness, measured as the variance in their response (Fig. 1a and 2a). The same situation occurs when females have unimodal preferences and share the same peak preference (Fig. 1e and 2e). However, when females show unimodal preferences and differ in their peak preferences (Fig. 1c), then they rank males differently in terms of attractiveness (Figs. 2c). If females differ in both peak preference and choosiness (Fig. 1g), then their rankings of male attractiveness can be startlingly variable (Fig. 2g), even though the population-level preference function for male attractiveness remains open-ended and similar to the population-level preferences for other models of mate choice (Fig. 2, right column). This exercise is mainly intended to visualize our intuition, and it shows that we can use preferences for male attractiveness to diagnose the shape of female preference functions without assuming that any particular male ornamental trait entirely determines attractiveness. In particular, we can cleanly distinguish cases where females show open-ended preferences or identical peak preferences (Figs. 2a and 2e) from those in which females vary in their peak preferences (Figs. 2c and 2g) based solely on individual-level variation in perceived male attractiveness.

Given that females likely vary in the different components of preference, an added nuance to the study of mate choice is that preferences can be studied at the level of either the individual or the population (Fig. 1). Arguably, population-level preferences are more easily characterized because their measurement does not require repeated observations of the same individuals. Population-level preferences are important from a sexual selection standpoint because they describe the selection surface for the intersexually selected traits of males (Wagner 1998; Chenoweth and Blows 2006; Jones *et al.* 2012). Of course, a population-level preference arises from the integration of individual-level preferences across the individuals comprising the population. In Fig. 1, we show five hypothetical individual-level female preference functions across ten evenly distributed male phenotypes in the left column and population-level preferences for the same ten males in the right-hand column. Fig. 1 demonstrates how population-level preference functions provide very little information regarding the underlying variation in individual-level functions. Nevertheless, these individual-level preferences take center stage when we begin to think about the evolution of the preferences themselves, because for a preference function to evolve, at least some aspects of the function must have a genetic basis. Unfortunately, the study of individual-level preferences brings with it a host of additional empirical hurdles. Two major issues stand out as being particularly problematic. The first is that the characterization of individual-level mating preferences requires repeated measurements of the same female. If a female's preferences change over time or if she becomes habituated to the preference assay, then her true preferences may be nearly impossible to measure. The second problem is that there may be no way to know *a priori* which ornamental traits of males are most important in terms of mating decisions. Thus, if key traits remain unmeasured, then individual-level preference functions may prove inaccessible. A potential solution to this problem is to focus on overall male attractiveness rather than individual traits (Fig. 2).
